# Influence of family and friend smoking on intentions to smoke and smoking-related attitudes and refusal self-efficacy among 9–10 year old children from deprived neighbourhoods: a cross-sectional study

**DOI:** 10.1186/s12889-015-1513-z

**Published:** 2015-03-07

**Authors:** Ciara E McGee, Joanne Trigwell, Stuart J Fairclough, Rebecca C Murphy, Lorna Porcellato, Michael Ussher, Lawrence Foweather

**Affiliations:** Centre for Public Health, Liverpool John Moores University, Henry Cotton Campus, 15-21 Webster Street, Liverpool, L3 2AT UK; Centre for Health Promotion Research, Leeds Beckett University, Calverley Building, City Campus, Leeds, LS1 3HE, UK; Department of Sport and Physical Activity, Edge Hill University, St. Helens Road, Ormskirk, Lancashire, L39 4QP UK; Physical Activity Exchange, Research Institute for Sport and Exercise Sciences, Liverpool John Moores University, 62 Great Crosshall Street, Liverpool, L3 2AT UK; Institution of Population Health Research, St George’s, University of London, Cranmer Terrace, London, SW17 0RE UK; Department of Physical Education and Sport Sciences, University of Limerick, Limerick, Ireland

**Keywords:** Smoking, Children, Mother smoking, Father smoking, Sibling smoking, Peer smoking

## Abstract

**Background:**

Smoking often starts in early adolescence and addiction can occur rapidly. For effective smoking prevention there is a need to identify at risk groups of preadolescent children and whether gender-specific intervention components are necessary. This study aimed to examine associations between mother, father, sibling and friend smoking and cognitive vulnerability to smoking among preadolescent children living in deprived neighbourhoods.

**Methods:**

Cross-sectional data was collected from 9–10 year old children (n =1143; 50.7% girls; 85.6% White British) from 43 primary schools in Merseyside, England. Children completed a questionnaire that assessed their smoking-related behaviour, intentions, attitudes, and refusal self-efficacy, as well as parent, sibling and friend smoking. Data for boys and girls were analysed separately using multilevel linear and logistic regression models, adjusting for individual cognitions and school and deprivation level.

**Results:**

Compared to girls, boys had lower non-smoking intentions (P = 0.02), refusal self-efficacy (P = 0.04) and were less likely to agree that smoking is ‘definitely’ bad for health (P < 0.01). Friend smoking was negatively associated with non-smoking intentions in girls (P < 0.01) and boys (P < 0.01), and with refusal self-efficacy in girls (P < 0.01). Sibling smoking was negatively associated with non-smoking intentions in girls (P < 0.01) but a positive association was found in boys (P = 0.02). Boys who had a smoking friend were less likely to ‘definitely’ believe that the smoke from other people’s cigarettes is harmful (OR 0.57, 95% CI: 0.35 to 0.91, P = 0.02). Further, boys with a smoking friend (OR 0.38, 95% CI: 0.21 to 0.69, P < 0.01) or a smoking sibling (OR 0.45, 95% CI: 0.21 to 0.98) were less likely to ‘definitely’ believe that smoking is bad for health.

**Conclusion:**

This study indicates that sibling and friend smoking may represent important influences on 9–10 year old children’s cognitive vulnerability toward smoking. Whilst some differential findings by gender were observed, these may not be sufficient to warrant separate prevention interventions. However, further research is needed.

## Background

Globally, between 82,000 and 99,000 young people start smoking every day [1]. Although the proportion of 8–15 year olds in the United Kingdom (UK) who have ever smoked has declined from 18.7% in 1997 to 6% in 2013 [2], over 200,000 start to smoke each year [3]. Smoking poses many health risks, including various forms of cancer, cardiovascular disease and respiratory disease, and imposes a significant financial and social burden on society [4]. Therefore smoking prevention remains an important public health priority [5]. Efforts to delay or prevent children from starting to smoke are needed because the earlier a child starts to smoke, the less likely they are to quit the habit as an adult, and the more likely they are to die prematurely from a smoking-related disease [6].

Primary school children represent an important cohort for smoking prevention as regular smoking is not yet established (0.3% ever smoked at age 8–10 years) [2]. Although these children do not smoke, they may have developed intentions regarding future smoking [7]. In accordance with the Theory of Planned Behaviour (TPB) [8], future intentions to smoke predict subsequent smoking behaviour [7,9]. In turn, intentions to smoke are shaped by an individual’s smoking-related cognitions such as attitudes (the overall evaluation of smoking) and self-efficacy expectations (a person’s confidence in their ability to stay a non-smoker and to refuse a cigarette) [8,10-12]. Research in adolescents has demonstrated that individual cognitions are formed by distal factors at the interpersonal level, such as family and peers [13,14]. Less is known about the factors that influence preadolescent children’s individual cognitions and such knowledge can be used to inform the development of smoking prevention interventions.

Bandura’s social learning theory (SLT) [15] postulates that smoking behaviour may be directly acquired through modelling the behaviour of significant others. Similarly, attitudes and values towards smoking are partly formed from observing others smoking [15]. In accordance with social learning theory, previous studies have shown parental, sibling and peer smoking to be significant risk factors for smoking uptake [16,17]. Previous research in US preadolescents has shown that having a family member that smokes is associated with more favourable implicit attitudes towards smoking compared with preadolescent children with non-smoking family members [18]. Similarly, research in Dutch preadolescent children found exposure to parental, sibling and peer smoking to be associated with having more pro-smoking attitudes [19]. Parental smoking was also related to perceived safety of casual smoking and temptation to smoke in response to smoking related cues such as seeing someone smoke [19]. Accumulative evidence suggests that there are gender differences concerning the influence of social factors on smoking uptake in adolescents [20,21]. For example, mother smoking is reported to influence smoking uptake in girls [20], whereas father and friend smoking have been found to be stronger influences for boys [22,23]. However, the influence of social factors on the antecedents of smoking behaviour in preadolescent boys and girls is less clear. Such knowledge may inform decisions surrounding the inclusion of gender-specific components in smoking prevention interventions targeted at preadolescent children.

Smoking is socially patterned, with high smoking prevalence among low socio-economic status (SES) groups [24]. This is important as smoking is the leading cause of health inequalities [25]. Addressing inequalities in tobacco use is therefore a public health priority [26] and socially deprived areas have been identified as an important target for smoking interventions [27]. SES is widely regarded as being an important determinant of smoking uptake in young people as children who live and go to school in socially deprived areas are more often exposed to smoking behaviour [24,28]. Given that children who live in deprived neighbourhoods are likely to include a predisposition to experiment with smoking [29], further insight into factors that influence smoking-related cognitions in these groups can provide additional knowledge to inform the development of interventions. A recent and large cross-sectional study of Dutch primary school children (aged 10–11 years) found that the smoking behaviour of the father, mother and other family members was shown to be the most influential on the intention to smoke among children living in a low SES area, though more evidence is needed [30].

To the authors’ knowledge, the only published UK study that has been conducted with preadolescent children is the Liverpool Longitudinal Study (LLSS) [31-33]. The city of Liverpool is one of five metropolitan boroughs in Merseyside, England, and is ranked among the most deprived local authorities in England [34]. In the LLSS study, 8% of nine year olds had tried smoking, with rates rising to 21% at age 10 and to 27% at age 11. Smoking experimentation was higher amongst boys at age 10, and factors associated with children’s smoking were parental and best friend smoking, curiosity, living in a low income family and residing in a deprived area. However, the LLSS was a largely qualitative study that included a small cohort of children from six primary schools in a localised area of Liverpool. Further, whilst the LLSS examined smoking uptake it did not examine factors associated with intentions to smoke and individual smoking-related cognitions, which are important from a primary prevention perspective.

This paper seeks to extend the LLSS by conducting a large quantitative study and involving a regional population of 9–10 year primary school children from two metropolitan boroughs in Merseyside. Further, the research aims to add to the limited evidence base of studies investigating the influence of social factors on outcomes relevant for primary prevention (i.e. before smoking use or experimentation), in particular among low SES populations. Therefore, the present study aimed to examine the association between social factors (mother/father/sibling/friend smoking) and intentions to smoke and individual smoking-related cognitions (attitude toward smoking, refusal self-efficacy expectations) among preadolescent children from socially deprived areas of the UK. The study investigated social influences on these aspects of cognitive vulnerability toward smoking by gender, as at present there is only limited understanding of the reasons behind gender patterns in smoking [35].

## Methods

### Participants and procedures

This cross-sectional study presents baseline data collected from a smoking prevention intervention study called ‘SmokeFree Sports’, between September-October 2012. SmokeFree Sports is a 7-month physical activity intervention, involving coach and teacher training and the provision of sports activities, to prevent smoking among 9–10 year old primary school children in Liverpool, a city in Merseyside, England. The intervention has been described in detail elsewhere [36] and will be evaluated within a non-randomised controlled trial. Since the funding for the project required that the intervention be offered to all schools in Liverpool, randomisation of local schools was not possible; therefore, prior to the recruitment of schools, Liverpool was matched with Knowsley, another metropolitan borough in Merseyside, on the basis of population data, including adult smoking rates (Liverpool: 24.2%; Knowsley: 27.6%) [37], deprivation level [38] and ethnic composition [39]. Children in the present study were therefore recruited through primary schools in Liverpool and Knowsley local authorities. Merseyside provides a unique context for the research as it has some of the most deprived local authorities in England [34]. Furthermore, the health of children and young people in Liverpool and Knowsley is worse than the England average [40,41]. Ethical approval for the study was granted by Liverpool John Moores University Research Ethics Committee (12/SPS/038).

In September 2012, all eligible primary schools (mainstream state schools; n = 154), from Liverpool (n = 104) and Knowsley (n = 50), were invited to participate in the study. Schools received information about the project via email and post. To enhance participation rates, schools who had not responded were followed-up with telephone calls. Following initial communication with each school, site visits were made by the research team to share information about the project with staff acting as study coordinators. Study information was passed on to senior staff members and written consent was requested if they wished their school to participate. In total, 43 schools agreed to take part in the study (28%), including 32 (31%) from Liverpool and 11 (22%) from Knowsley. Schools that declined to participate provided diverse reasons for not taking part (e.g., too busy, key teacher on sick leave, already in receipt of external projects). In participating schools, all Year 5 children (aged 9–10 years; n = 1393) were invited to take part. This age group was chosen because by age 11 almost one quarter of children will have tried smoking [42]. Furthermore, whilst it is not mandatory to address smoking education in Key Stage 2 (pupils aged 7 to 11) of the UK National Curriculum [43], the National Institute for Health and Care Excellence (NICE) [44] postulates that smoking prevention efforts would be most effective if they began in primary school.

To recruit children, the ethics committee gave approval for a passive informed consent procedure with parents/guardians provided with an opportunity to opt out of the study if they did not want their child to participate. Specifically, schools were given a stamped addressed envelope containing a participant information sheet and opt-out form to mail to parents. Parents could opt their child out of the study by signing and returning the opt-out form or calling the research team. Following an opt-out deadline of at least two weeks, schools were visited to obtain child assent and collect baseline data. Parental consent and child assent were obtained for 1339 children (96% response rate). During data collection, 123 children were absent from class. Children were excluded from the study if they had a special class placement (e.g., learning disability), difficulty in speaking and or understanding the English language (n = 33), or incomplete outcome measures (n = 17). The smoking questionnaire was completed on school laptop computers using a web-based survey (www.surveymonkey.com). A member of the research team stood at the front of the class and guided children through the questionnaire and read questions aloud as required by children. To aid true and accurate responses, questionnaires were completed in silence and confidentiality was stressed to all participants. The online survey took children approximately 30 minutes to complete. Completed surveys were submitted by each child and responses were immediately transmitted to a secure electronic database for subsequent analysis.

### Measures

#### Smoking questionnaire

A questionnaire was constructed using items adapted from questionnaires previously used with this age group [45-48]. Demographic information measured included age (years), gender (0 = boy; 1 = girl), ethnicity (1 = White British, 2 = White non-British, 3 = Mixed ethnicity, 4 = South Asian – Indian/Pakistani/Bangladeshi, 5 = Black – African/Caribbean/British, 6 = Chinese, 7 = other non-British descent, e.g. Arab) and SES. Home postcodes, provided by the children, were used to estimate SES. Postcode data was entered into ‘GeoConvert’ [49], a free online tool that generates indices of multiple deprivation (IMD) scores. IMD scores are a composite of seven domains of deprivation (income, employment, education, health, crime, access to services, and living environment) [50], with higher scores representing higher degrees of neighbourhood deprivation and therefore lower SES. Individual level outcome measures included intention to smoke and smoking-related cognitions such as refusal self-efficacy and attitudes toward smoking (collectively termed ‘cognitive vulnerability toward smoking’). Parent, sibling and friend smoking behaviour were assessed to examine the influence of social factors. Child smoking behaviour was measured for descriptive purposes using a single item from the Health Survey for England [48]. Children were asked to indicate which of five stages of smoking best described them, from (1) ‘I have never smoked, not even one puff’ to (5) ‘I smoke at least once a day’. Responses were re-coded to ‘never tried smoking (not even one puff)’ (0), and ‘tried smoking’ (any experimentation with smoking) (1). As an indicator of smoking status, expired carbon monoxide (CO) concentrations were taken in private and recorded using a piCOsimple Smokerlyzer (Bedfont Scientific UK, England) with a reading above 10 ppm used as cut-off for defining smokers [51].

### Individual cognitive vulnerability to smoking

Intention (not) to smoke was assessed using two items from the Health Survey for England [48], ‘Do you think you will smoke in the next month/year?’, as well as an item designed by the research team ‘Do you think you will smoke in secondary school?. Responses ranged from ‘definitely yes’ (1) to ‘definitely not’ (4) and were summed to produce a total intention score (range 3–12). A high score on total intention indicated a strong intention not to smoke. Cronbach alpha for total intention showed good internal consistency (α = 0.81).

Refusal self-efficacy was measured using three items adapted from a nine-item self-efficacy scale in adolescents [45]. Pilot work with children indicated that the question and answer formats used within these items were developmentally inappropriate for 9–10 year olds and therefore each item was amended to reflect this age level. Items assessed the child’s confidence in their ability to be a non-smoker and refuse cigarettes in different situations: ‘How confident are you that you can stay (become) a non-smoker?’ ‘How confident are you that you could say no to a cigarette if someone offered you one?’ and ‘How confident are you that you could be a non-smoker if your friends smoke?’ Responses consisted of Likert scales ranging from ‘not confident at all’ (1) to ‘very confident’ (5) and were summed to create a total refusal self-efficacy score (range 3–15). Cronbach alpha for the combined scale showed good internal consistency (α = 0.81). A high score on the scale indicates a high level of refusal self-efficacy.

Attitude structure includes affective, behavioural and cognitive components [52]. For the purpose of this study, children’s beliefs and knowledge about smoking were explored through the cognitive component of attitudes adapted from the Global Youth Tobacco Survey (GYTS) [47] and the Health Survey for England [48], including ‘Do you think smoking is bad for your health?’, ‘Once someone has started smoking, do you think it will be difficult to quit?’, ‘Do you think that it is safe to smoke for only a year or two as long as you quit after that?’, ‘Do you think the smoke from other people’s cigarettes is harmful to you?’. An additional item ‘Do you think smoking effects sport performance?’ was developed by the research team. Responses ranged from ‘definitely not’ (1) to ‘definitely yes’ (4). A summary scale was created but internal consistency was low (α = .49). Since the data for individual attitude items were positively skewed and distribution was not improved by statistical transformation, responses were collapsed into dichotomous variables for analyses: a definitive negative attitude towards smoking (i.e. ‘definitely yes’) was scored 1; the remaining response categories (i.e. ‘probably yes’, ‘probably not’ and ‘definitely not’) indicated a more favourable attitude towards smoking and thus were collapsed into a single group and scored 0. One attitude item (‘Do you think that it is safe to smoke for only a year or two as long as you quit after that?’) was reverse coded in order to maintain consistent scale direction for all items. An additional attitude item, ‘Do you think smoking makes you gain weight?’ was also included from the Health Survey for England [48]. Responses for this item were collapsed into a dichotomous variable for analysis with ‘no difference’ scored 1 and the remaining response categories (i.e., ‘lose weight’ or ‘gain weight’) grouped and scored 0.

### Parent, sibling and friend smoking behaviour

Perceived parent and sibling smoking behaviour were assessed using an item taken from the Health Survey for England [48]. Children were asked to select who in their family smokes from nine items (e.g., mum, step-mum, brother, uncle, cousin), and could enter additional family members who smoke if necessary. Since this study was concerned with the influences of immediate family members, only (biological) mother, father and sibling smoking behaviours were used in the analyses. Children with a smoking mother/father/sibling were scored 1. Children with a non-smoking mother/father/sibling were scored 0. Perceived friend smoking was assessed using two items adapted from an existing survey [46] ‘Do any of your friends smoke?’ and ‘Have any of your friends tried smoking?’ Responses were 1 = ‘none of my friends’, 2 = ‘a few of my friends’, 3 = ‘most of my friends’, 4 = ‘all of my friends’. For subsequent analysis, items and responses were collapsed to create the dichotomous variable of: ‘friends had not tried smoking (friends do not smoke and have not tried smoking; scored 0) or ‘friends smoke’ (friends smoke or had tried smoking; scored 1).

### Analyses

Descriptive statistics were calculated for the sample and by gender and reported as means (±SD) or proportions (%). Gender differences in means were examined using independent t-tests, with categorical variables tested using chi-square tests of association. Multilevel linear and logistic regression analyses were conducted to examine continuous variables and dichotomous outcome measures, respectively. To account for children being nested in schools, a 2-level data structure was used. Children were defined as the first level unit of analysis, and school was the second level unit of analysis. Separate analyses were conducted for boys and girls to assess associations between mother, father, sibling and friend smoking and intentions to smoke and smoking-related cognitions (i.e., refusal self-efficacy and attitudes toward smoking), adjusting for deprivation level. Each model was adjusted for other individual level cognitive variables (e.g. for the intention model, adjustments were also made for refusal self-efficacy scale and dichotomous attitude items) since these variables may influence each other [8,15]. Regression coefficients in each model were assessed for significance using the Wald statistic. Analyses were performed using MLwiN 2.30 software (Centre for Multi-level Modelling, University of Bristol, UK) with statistical significance set at P < 0.05.

## Results

Descriptive statistics and gender differences for the study sample (n = 1143; Mean age: 9.6 years, SD 0.3; 49.3% boys; 82% participation rate) are presented in Table 1. A high proportion of the children were white British (85.6%), with the remaining children self-identified as black (4.1%), white non-British (1.6%), mixed race (2.8%), Asian (2.6%), Chinese (0.8%) or other non-British descent (2.5%). Over eight out of ten participating children lived within an area ranked within the top 20% for deprivation in England, with 75% within the most deprived decile [50]. The majority of children (97.5%) reported to have never smoked. CO readings were recorded for 82.7% of children (n = 945). Children’s self-reported non-smoking was confirmed by CO readings (Mean = 1.3, SD ±0.7), with all participants readings below 10 ppm. Children’s perceived smoking behaviour of family and friends is also shown in Table 1. Over half of children (57.3%) reported having at least one family member who smokes; 37.1% mothers, 39.0% fathers and 11.0% of siblings were current smokers. Around a sixth of children had at least one friend who smokes.

**Table 1:** **Descriptive characteristics for the study participants**

	**All**	**Boys**	**Girls**	**P value**
	**(n = 1143)**	**(n = 563)**	**(n = 580)**	
	**M ± SD or %**	**M ± SD or %**	**M ± SD or %**	
**Demographics**				
Age (years)	9.6 ± 0.3	9.6 ± 0.3	9.6 ± 0.3	0.06
Ethnicity (White British)	85.6	86.1	85.0	0.75
Deprivation level (IMD)	54.8 ± 16.8	54.4 ± 16.7	55.2 ± 16.9	0.42
**Social influences**				
Mother smoking	37.1	35.1	39.0	0.18
Father smoking	39.0	39.3	38.8	0.87
Sibling smoking	11.0	9.9	12.1	0.25
Friend smoking^†^	16.4	21.7	11.2	<0.01*
**Smoking intentions**				
Total non-smoking intentions (range 4-12)	11.7 ± 0.9	11.6 ± 1.0	11.8 ± 0.7	0.02*
**Self-efficacy**				
Total refusal self-efficacy (range 3-15)	13.6 ± 3.1	13.4 ± 3.3	13.8 ± 3.0	0.04*
**Attitudes towards smoking**				
Smoking is bad for health (‘*definitely yes’*)	88.8	85.4	92.1	<0.01*
Safe to smoke year or two (‘*definitely not’*)	62.6	62.5	62.8	0.93
Difficult to quit once started (‘*definitely yes’*)	50.7	50.4	51.0	0.84
Others smoke harmful to you (‘*definitely yes’*)	64.3	62.5	66.0	0.22
Effects sports performance (‘*definitely yes*’)	55.8	56.8	54.8	0.49
Makes you gain or lose weight (‘*no difference’*)	42.1	43.9	40.3	0.23

Notes: IMD, Indices of multiple deprivation score; ^(†)^ at least one friend smokes/tried. Independent t-tests and chi-square statistics were used to determine differences in means and percentages, respectively. *Significant gender difference (P < 0.05).

Whilst a high proportion of children (88.8%) agreed that smoking is ‘definitely’ bad for health, more favourable attitudes towards smoking were observed for the remaining attitude items (Table 1). Approximately six out of ten children indicated that they ‘definitely’ agreed that: ‘it is not safe to smoke for a year or two as long as you quit after that’, ‘the smoke from other people’s cigarettes is harmful to you’ and that ‘smoking effects sports performance’. Further, only half of children believed that it is ‘definitely’ difficult to quit smoking once started, whilst almost six out of ten children stated that smoking makes you either gain or lose weight. Gender differences are also shown in Table 1. Compared to girls, boys had lower non-smoking intentions (P = 0.02) and refusal self-efficacy (P = 0.04). In addition, boys reported having more smoking friends (P < 0.01), whilst a higher proportion of girls than boys believed that smoking is ‘definitely’ bad for health (×^2^ = 12.6, P < 0.01, phi = .10). No other sex differences were observed.

### Non-smoking intentions

Table 2 shows associations between social factors and non-smoking intentions. After adjustment for refusal self-efficacy, attitudes towards smoking and school and deprivation level, friend smoking was negatively associated with non-smoking intentions in both boys (P < 0.01) and girls (P < 0.01); sibling smoking was negatively associated with non-smoking intentions in girls (P < 0.01) but a positive association was found in boys (P = 0.02). Neither mother nor father smoking behaviour was associated with non-smoking intentions.

### Refusal self-efficacy

Table 2 also shows associations between social factors and refusal self-efficacy. After adjustment for non-smoking intentions, attitudes towards smoking and school and deprivation level, friend smoking was negatively associated with refusal self-efficacy in girls (P < 0.01) but not boys (P = 0.07). Neither mother, father nor sibling smoking was associated with refusal self-efficacy.

**Table 2:** **Summary of multilevel regression analysis examining associations between social factors and non-smoking intentions and refusal self-efficacy**

	**Non-smoking intentions**		**Refusal self-efficacy**	
	**β (95% CI)**	**P value**	**β (95% CI)**	**P value**
**Boys**				
Mother smoking	-0.03 (-0.20, 0.14)	0.70	-0.40 (-0.98, 0.18)	0.18
Father smoking	0.02 (-0.15, 0.18)	0.86	-0.25 (-0.74, 0.25)	0.33
Sibling smoking	0.32 (0.05, 0.60)	0.02*	-0.49 (-1.33, 0.36)	0.26
Friend smoking^†^	-0.57 (-0.77, -0.37)	<0.01*	-0.57 (-1.18, 0.04)	0.07
**Girls**				
Mother smoking	-0.04 (-0.15, 0.08)	0.53	-0.02 (-0.52, 0.49)	0.94
Father smoking	-0.01 (-0.13, 0.10)	0.81	-0.32 (-0.81, 0.17)	0.19
Sibling smoking	-0.38 (-0.55, -0.21)	<0.01*	0.43 (-0.33, 1.19)	0.26
Friend smoking^†^	-0.33 (-0.49, -0.17)	<0.01*	-1.14 (-1.86, -0.42)	<0.01*

Notes: β, Beta coefficient; CI, confidence interval; ^(†)^ at least one friend smokes or tried. Beta (95% CI) values reflect the associations between mother, father, sibling and friends smoking and (a) non-smoking intentions or (b) refusal self-efficacy. All models were adjusted for school and deprivation level; non-smoking intention models were also adjusted for refusal self-efficacy and attitudes towards smoking; refusal-self-efficacy models were also adjusted for non-smoking intentions and attitudes towards smoking. *Significant association (P < 0.05).

### Attitudes towards smoking

Table 3 presents associations between social factors and children’s attitudes towards smoking, after adjustment for non-smoking intentions, refusal self-efficacy and school and deprivation level. Significant associations were observed for social factors and attitudes toward smoking on two out of six attitude items for boys; however, no associations were found in girls. Compared to boys with non-smoking friends, boys with smoking friends were less likely to ‘definitely’ believe that smoking is bad for your health (Odds Ratio (OR) = 0.38, 95% CI: 0.21 to 0.69, P < 0.01) and the smoke from other people’s cigarettes is harmful to you (OR = 0.57, 95% CI: 0.35 to 0.91, P = 0.02). In comparison to boys with a non-smoking sibling, boys with a smoking sibling were less likely to ‘definitely’ believe that smoking is bad for your health (OR = 0.45, 95% CI = 0.21 to 0.98, P = 0.04). Mother, father and sibling smoking were not associated with any attitude items in boys or girls.

**Table 3:** **Summary of multilevel binary logistic regression analysis for social factors associated with children’s attitudes towards smoking**

**Attitude item**	**Bad for health**		**Not safe to smoke year or two**		**Difficult to quit once started**		**Others smoke is harmful to you**		**Effects sports performance**		**Makes no difference to your weight**	
**Predictor**	**OR (95% CI)**	**P value**	**OR (95% CI)**	**P value**	**OR (95% CI)**	**P value**	**OR (95% CI)**	**P value**	**OR (95% CI)**	**P value**	**OR (95% CI)**	**P value**
**Boys**												
Mother smoking	0.87 (0.50,1.54)	0.64	0.69 (0.47, 1.01)	0.05	0.75 (0.51, 1.09)	0.13	1.18 (0.78, 1.80)	0.44	1.36 (0.91, 2.04)	0.14	1.28 (0.88, 1.85)	0.19
Father smoking	0.68 (0.39, 1.17)	0.16	1.18 (0.82, 1.71)	0.37	0.96 (0.67, 1.37)	0.81	1.15 (0.77, 1.73)	0.48	1.02 (0.69, 1.50)	0.92	0.91 (0.64, 1.29)	0.59
Sibling smoking	0.45 (0.21, 0.98)	0.04*	0.95 (0.52, 1.76)	0.88	1.11 (0.60, 2.05)	0.74	1.85 (0.93, 3.69)	0.08	0.67 (0.35, 1.28)	0.23	1.19 (0.66, 2.14)	0.57
Friend smoking	0.38 (0.21, 0.69)	<0.01*	0.73 (0.47, 1.15)	0.18	1.13 (0.83, 2.08)	0.24	0.57 (0.35, 0.91)	0.02	1.07 (0.66, 1.72)	0.80	0.65 (0.42, 1.02)	0.06
**Girls**												
Mother smoking	1.09 (0.52, 2.20)	0.82	0.69 (0.47, 1.02)	0.07	0.86 (0.59, 1.26)	0.44	0.81 (0.53, 1.24)	0.32	1.18 (0.78, 1.77)	0.43	1.13 (0.78, 1.64)	0.53
Father smoking	0.82 (0.41, 1.64)	0.58	1.00 (0.68, 1.47)	1.00	1.29 (0.89, 1.86)	0.18	1.33 (0.87, 2.03)	0.18	0.68 (0.46, 1.01)	0.05	1.12 (0.78, 1.60)	0.54
Sibling smoking	1.69 (0.55, 5.15)	0.36	1.75 (0.95, 3.21)	0.07	0.84 (0.47, 1.49)	0.55	1.04 (0.55, 1.95)	0.90	0.71 (0.39, 1.29)	0.26	0.92 (0.57, 1.47)	0.73
Friend smoking	1.30 (0.49, 3.46)	0.60	0.77 (0.44, 1.33)	0.35	0.85 (0.50, 1.47)	0.57	1.32 (0.72, 2.43)	0.36	0.96 (0.54, 1.69)	0.88	1.11 (0.65, 1.88)	0.71

Notes: OR, odds ratio; CI, confidence interval. OR (95% CI) values reflect the strength of association between mother, father, sibling or friend smoking on attitudes towards smoking. All models were adjusted for non-smoking intentions, refusal self-efficacy, and school and deprivation level. *Significant association (P < 0.05).

## Discussion

The aim of the present study was to identify whether mother, father, sibling and friend smoking were associated with cognitive vulnerability to smoking among 9–10 year old children from deprived neighbourhoods in Merseyside, England. The results indicate that sibling and friend smoking may represent more salient influences on children’s cognitive vulnerability to smoking than mother and father smoking. Moreover, some differential effects were observed by gender, suggesting that social factors may, in part, influence the antecedents of smoking behaviour in boys and girls differently. These findings extend the LLSS [31-33] and add to the limited evidence base in preadolescent children.

SLT proposes that behaviour, perceptions of behaviour and the environment interact to influence one another [15]. In accordance with SLT [15], parents have previously been considered to be the most important influences on children during the primary school years [53], while peer influences become increasingly more salient during the adolescent years [54]. In the present study, mother (37%) and father smoking (39%) was relatively high, which is reflective of the local context in Merseyside where levels of smoking and deprivation are higher than the national average [4,38]. Children of smoking parents are at a higher risk of having susceptible smoking cognitions [12,18,19,54-56] and initiating smoking [16], especially those in lower socio-economic status groups [30]. However, in the current study, no associations were observed between mother or father smoking and children’s non-smoking intentions, smoking-related attitudes and refusal self-efficacy. A possible explanation for the divergence in findings is that whilst this study examined independent influences of mother and father smoking, other studies [12,18,19,54-56] utilised a combined parental smoking variable for analyses. To check this, we conducted additional analysis using a combined parental smoking variable but found no further associations. Alternatively, whilst children are aware that their parents smoke, their exposure to smokers may vary [19] as a result of regional public health campaigns to protect children from smoking such as “Take 7 Steps Out” (see www.tobaccofreefutures.org). In addition, smoking parents may communicate non-smoking expectations to their offspring or display disapproval of child smoking, which has been found to be protective against smoking intention and initiation [16,30,53,57,58]. Nevertheless, further research is needed to examine the influence of mother or father smoking behaviour on children’s cognitive vulnerability towards smoking.

The results of the present study suggest that sibling and friend smoking may be important influences on preadolescent children’s cognitive vulnerability towards smoking. Friend smoking was negatively associated with non-smoking intentions in both boys and girls, extending previous studies in adolescents that have found peer smoking to be related to smoking uptake [17]. The influence of sibling smoking, however, differed by gender; sibling smoking was negatively associated with non-smoking intentions in girls, which is consistent with the accumulative evidence [16]. Conversely, a positive association was apparent in boys, suggesting that having a smoking sibling strengthened their non-smoking intentions. This finding was unexpected but may reflect parent disapproval of sibling smoking and communication of non-smoking expectations [53,57], although more research is needed. Gender differences were also found in relation to refusal self-efficacy and attitudes toward smoking; friend smoking was negatively associated with refusal self-efficacy in girls but not boys. Further, boys with a smoking friend or sibling had less negative attitudes towards smoking regarding the health consequences of smoking and the harms of others’ smoke though no associations were observed in girls. Boys reported having more smoking friends than girls, which may have contributed to these effects since children who perceive that many of their friends advocate or engage in smoking are more likely to develop pro-smoking attitudes [19]. Further, boys may assume that smoking is not as harmful, otherwise their friend/sibling would not smoke.

To the authors’ knowledge, only one other study has concurrently examined the role of parent, sibling and friend smoking in shaping preadolescents cognitive vulnerability to smoking [19]. Using structural equation modelling, Schuck et al. [19] found no direct effects of parental smoking, sibling smoking or peer smoking on 9–12 year old children’s susceptibility towards smoking. However, peer, sibling and, in particular, parent smoking was associated with perceiving more pros of smoking. Further, parent smoking was positively associated with perceived safety of casual smoking and cue-triggered wanting to smoke [19]. These findings are inconsistent with the current study and may reflect cultural differences and different methodologies employed. Future studies examining the influence of the social environment in preadolescents are warranted.

The findings observed for friend and sibling smoking on children’s cognitive vulnerability to smoking could be attributed to several factors. Firstly, while children in the early primary school years are likely to spend a lot of time with their parents, it is probable that older children (ages 8 years and over) spend more time with siblings (who share more similarities and social networks) and friends. The findings may therefore reflect the fact that friends and siblings increasingly represent children’s predominant social environment, and are likely to be more proximal influences on children’s vulnerability to smoking than parents. Second, peer and sibling smoking behaviour is likely to be less overt than parent smoking and as a consequence may be perceived by other children as exciting or cool and socially desirable [59]. Peer groups are known to share common attitudes and behaviours [60,61]; smokers may communicate pro-smoking attitudes and approval of smoking initiation [62], which in turn could influence intentions to smoke and smoking-related cognitions among children. Third, whilst the majority of children stated that they had never tried smoking (97.5%), around a sixth believed that they knew a friend that had. It is possible that children may have underreported their own smoking status, or perhaps, overestimated their friends smoking habits. Given that overestimation of smoking prevalence is related to smoking initiation in preadolescent children [63], overestimation of friend and sibling smoking by children in the current study may have influenced their cognitive aspects around smoking. Taken together, the results suggest that friend and sibling smoking behaviours may contribute to preadolescent children’s cognitive vulnerability to smoking. However, more evidence is required and research is needed to determine the mechanisms associated with peer and sibling influence.

Encouragingly, most children displayed strong non-smoking intentions and refusal self-efficacy. Reflecting the high intention not to smoke, few children had tried smoking (2.5%), which is consistent with other studies in preadolescent children [31,32]. NICE guidance [44] states that smoking prevention efforts may be more effective if started in primary school. Given the low rates of smoking experimentation, 9–10 year old children could be an appropriate cohort to target for primary prevention. While encouraging, results regarding children’s high refusal self-efficacy should be interpreted with caution because children at this age may not have encountered situations where they have been put to the test to resist influences to smoke from others [55]. Because decreases in self-efficacy have been associated with smoking onset and continuation in adolescents [64,65], efforts to maintain the strength of preadolescent children’s smoking refusal self-efficacy may be effective in preventing them from starting to smoke. Previous school-based interventions that have taught adolescents to deal with direct pressure to smoke have demonstrated modest positive results on smoking behaviour [66,67]. Prevention interventions may also need to address children’s attitudes toward smoking, as over a third of participants in this study did not recognise with certainty that short term smoking is not safe, that smoking is addictive, that others smoke is harmful, that smoking effects sport performance and that smoking per se does not influence weight. More positive attitudes toward smoking may predict intentions to smoke in the future and later smoking behaviour [8,10-12].

Previous research has called for further investigations into the need for gender-specific approaches to prevent smoking [30]. The current study found gender differences in the influence of social factors. In addition, compared with girls, boys were less likely to believe smoking is ‘definitely’ bad for health, and expressed lower non-smoking intentions and refusal self-efficacy. However, no clear pattern emerges from the data and qualitative research may prove useful in revealing the thought processes through which boys and girls form these smoking-related cognitions. Previous research with Dutch preadolescent children has reported it unnecessary to develop separate smoking prevention programmes for preadolescent children [64]. Given that the influences on boys’ and girls’ intentions to smoke were broadly similar, the results of the present study provide tentative support to this statement. Nevertheless, intervention and prevention efforts aimed at preadolescents may benefit from tailored messaging that dispels myths about the health consequences of smoking and exposure to smoke as well as strengthening refusal self-efficacy.

This study extends the smoking literature in preadolescent children by examining the influence of social factors (mother, father, sibling and friends) on cognitive vulnerability to smoking among a large sample of 9–10 year old children from deprived neighbourhoods. However, the study has a number of limitations. First, the analysis is based on a self-reported cross-sectional survey; therefore causal relationships cannot be established. In addition, the study examined influences on intentions to smoke and smoking-related cognitions, which may or may not result in smoking initiation at a later age [30]. Nevertheless, previous research demonstrates that these individual level factors are predictive of future smoking behaviour [8,10-12]. Second, children self-reported their smoking behaviour, which introduces the possibility of under or over reporting because of recall or social desirability [55]. However, self-reported smoking has been demonstrated to be accurate provided confidentiality is assured [68]. Moreover, children’s self-reported non-smoking status was confirmed using an objective measure of smoking. Third, direct measures of parental and friend smoking behaviours were not available, though previous research has demonstrated that children can reliably assess the smoking behaviour of others in their social environment [69]. Fourth, this study only examined the influence of biological family members (mother, father and sibling) and did not assess the influence of parental structure (i.e. one-parent vs. two-parent families or step parents). Previous research has shown adolescents who live with both biological parents smoke less than those living in single-parent families [70]. In addition, we did not collect gender-specific data on sibling smoking and therefore could not distinguish between the influence of brothers or sisters on the outcome variables. Finally, results are drawn from two deprived local authorities with high adult smoking prevalence, which limits the generalisability of results to other regions of England. However, given that smoking is socially patterned, findings can be generalised to similar urban areas with high levels of deprivation, where the need for smoking prevention is proportionally greater.

## Conclusions

In summary, the present study showed that whilst the majority of 9–10 year old children living in deprived communities had high non-smoking intentions and refusal self-efficacy, a substantial proportion displayed pro-smoking attitudes that could be addressed through smoking prevention efforts. Findings showed that social factors were associated with children’s cognitive vulnerability toward smoking, with the smoking behaviour of siblings and friends being identified as important influences. Whilst some differential findings by gender were observed, these may not be sufficient to warrant separate intervention approaches. This knowledge may aid the development of future smoking prevention interventions, though further research is needed.
